# Fluctuation in salivary alpha-amylase activity and vital signs during dental implant surgery

**DOI:** 10.1186/s40729-021-00339-6

**Published:** 2021-06-29

**Authors:** Afnan Sabbagh, Hidemi Nakata, Ahmed Abdou, Shohei Kasugai, Shinji Kuroda

**Affiliations:** 1 Department of Oral Implantology and Regenerative Dental Medicine, Division of Oral Health Sciences, Graduate School of Medical and Dental Sciences, Tokyo Medical and Dental University, 1-5-45 Yushima, Bunkyo-ku, Tokyo, 113-8510 Japan; 2 Department of Cariology & Operative Dentistry, Graduate School of Medical and Dental Sciences, Tokyo Medical and Dental University, 1-5-45 Yushima, Bunkyo-ku, Tokyo, 113-8549 Japan

**Keywords:** Salivary alpha-amylase, Stress, Patients, Anxiety, Implant surgery, Stress hormone, Vital signs

## Abstract

**Background:**

Salivary alpha-amylase (sAA) activity is thought to be an indicator of mental stress. However, the relationship between sAA activity and mental stress in patients undergoing dental implant treatment has not been investigated. The present study aimed to examine the correlation between sAA activity and changes in the patient’s vital signs during dental implant surgery.

**Results:**

sAA activity was higher after surgery compared to the pre-surgical measurements. A significant positive correlation was observed between sAA activity and heart rate (HR) (*r*_*s*_=0.434, *p*=0.007), as well as the peripheral blood oxygen (SpO_2_) (*r*_*s*_=0.392, *p*=0.016)*.*

**Conclusion:**

sAA activity and the patient’s stress levels tended to increase after the surgical procedure. A positive correlation was observed between SpO_2_ and sAA activity. A significant positive correlation was also observed between the between the HR and sAA activity, although there was no correlation between blood pressure and sAA activity. Thus, sAA may be a valuable indicator of the stress and anxiety accumulated during dental implant surgery.

## Background

Oral rehabilitation with dental implants is a widely used clinically effective and predictable modality for patients with complete or partial edentulism [1–3]. In addition to the cornerstone implant placement surgery, dental implant treatment requires different surgical techniques, such as bone augmentation, soft tissue management, or a second surgery to deliver the healing abutment, depending on each individual case [4]. These procedures may induce psychological and physiological stress in patients, which can be controlled in several cases, so that patients exhibit normal clinical conditions before and during surgical implant treatment [5, 6]. However, mental astress, such as sadness, anger, uneasiness or fear, and nervousness, can cause an elevation in blood pressure (BP) (i.e., hypertension), which can result in the cancellation of implant surgeries [7, 8]. Moreover, the surgical procedure may be cancelled owing to the incidence of a hypertensive episode immediately before the commencement of the procedure, despite the pre-surgical measurements being normal and a negative history of medical conditions. Unforeseen sudden cancellations are inconvenient to both patients and clinicians; upon enquiry, patients mention that they were anxious before surgery [9].

BP is controlled by the autonomic nervous system, which includes the sympathetic nervous system (SNS) and parasympathetic nervous system (PSNANS); in other words, the stress level in an individual is modulated by the balance between the PSNANS and SNS [10, 11].

Increasing stress levels induce SNS predominance, preparing the body for the “fight or flight” response, thereby increasing BP by increasing the heart rate (HR) and inducing contraction of the peripheral blood vessels.

Since anxiety is a form of psychological stress, an increase in anxiety results in a surge in stress levels [12, 13]. The human body reacts automatically in response to stress. Therefore, evaluating anxiety and/or stress levels in patients would be useful to ensure patient safety during treatment. Various questionnaires have been used to assess the degree of anxiety that have been shown to have simple application in routine clinical practice.

Cortisol levels in the peripheral blood have been used to evaluate stress levels, since the increase in stress promotes the secretion of cortisol from the adrenal glands [14, 15]. Furthermore, studies have reported that alpha-amylase activity in the saliva also increases with the increase in stress [16, 17]. The measurement of salivary alpha-amylase (sAA) activity is currently possible within less than 1 min, whereas the measurement of cortisol requires more time. Thus, the measurement of sAA would be advantageous for evaluating stress levels in patients in routine dental practice.

One study showed that sAA activity increased significantly when patients were exposed to a stressful condition compared to a relaxed condition [18]. Another study reported a significant correlation between the State/Trait Anxiety Inventory score and alpha-amylase levels [19].

The present study aimed to examine the anxiety and stress levels of patients who underwent dental implant surgery based on the activity of sAA before and after the procedure, in addition to the evalution of their vital signs. The purpose of this study was to examine the correlation between sAA activity, as an efficient stress biomarker for anxiety, and stress levels, as measured by the changes in the vital signs, such as BP, HR, and oxygen level, in patients undergoing dental implant surgery. The measurement of sAA activity acted as an adjunctive indicator for stress and anxiety and we assessed the relationship and correlation between sAA and these vital signs to test this hypothesis.

## Materials and methods

### Design and setting

This clinical study incorporated a prospective, non-controlled, non-interventionist design and was conducted at Tokyo Medical and Dental University’s Dental Hospital. Patients were recruited from the outpatient clinic.

### Ethical considerations

This study was approved by the ethics committee of Tokyo Dental and Medical University (approval numbers: D2020-027 and D2020-028).

### Participants

A total of 44 patients who underwent surgery for dental implant placement were recruited, including 28 women and 16 men. The participants’ mean age was 62 years. We divided patients into the following (two) main groups, depending on the type of implant surgery: the first surgery (*n*= 19) and second implant surgery(*n*=22) groups. Only three patients underwent connective tissue graft placement. However, the final sample size was reduced to 40 patients, as three patients were excluded because of incorrect data and the fourth patient cancelled the procedure due to a hypertensive crisis.

The exclusion criteria for this study were as follows: (1) uncontrolled diabetes, (2) pregnancy or nursing, (3) substance abuse, (4) very heavy smoking habit (≥ 11 cigarettes/day), (4) treatment with intravenous aminobisphosphonates, (5) severe bruxism, (6) untreated periodontitis, (7) poor oral hygiene, and (8) untreated mental disorders. The purpose of the study was explained to all patients, and consent was obtained.

Salivary amylase activity and vital sign measurement Salivary amylase activity was measured using a handheld salivary amylase monitor (NIPRO, Osaka, Japan). This analyzer enables automatic and time-efficient measurement of salivary amylase activity using a dry-chemical system, within approximately 1 min from the collection of saliva to the completion of measurement. The tip of the testing strip was placed under the tongue for 30 s to collect a sufficient amount of saliva so that it would completely cover the testing strip. Thereafter, it was removed from the mouth, and the other end of the tip was pulled slowly until it clicked and the testing strip was immediately inserted into the device. The plastic arm was pulled upwards, setting off a 10-s countdown on the device’s screen. Subsequently, the testing strip was pulled partially until it clicked again, and the result was displayed on the screen after an additional 10 s.

The tip of the testing strip was soaked in a buffer containing 2-chloro-4-nitrophenyl-4-O-beta-D-galactopyranosylmaltoside (Gal-G2-CNP), which acts as a substrate and chromogen, and dried. One-unit activity (U) per mass of enzyme is defined as the amount of enzyme whose activity produces 1 μmol of the reducing sugar maltose in 1 min [20].

We also measured all vital signs of all patients with the hospital monitor [HR, BP (Table 1), peripheral blood oxygen saturation (SpO_2_), and body temperature] at three time-points: (1) before, (2) during, and (3) after surgery.

**Table 1:** Reference values of blood pressure (mmHg)

Normal	Less than 120/80
Elevated	120–129/80
High (stage 1)	130–139/80–89
High (stage 2)	≥140/≥90
Hypertensive crisis	>180/>120

The first sAA sample was obtained and analyzed immediately before surgery, i.e., prior to the administration of local or intravenous anesthesia. Another sample was obtained immediately after the completion of surgery and before the patient rinsed his/her mouth.

### Statistical analysis

The Kolmogorov–Smirnov test was used to assess the normality of data distribution. The preoperative and postoperative amylase and SpO_2_ values were compared using the Wilcoxon signed-rank, owing to their non-parametric distribution. The preoperative and postoperative BP and HR values were compared using the paired t-test, owing to their parametric distribution. The differences between the preoperative and postoperative values of all variables were calculated, and Spearman’s correlation was performed to assess the correlation between the different variables. Statistical analysis was conducted using the IBM SPSS Statistical Software version 23 (Armonk, USA).

## Results

Table 2 summarizes the descriptive statistics for the entire study sample.

**Table 2:** Descriptive statistics for the outcomes investigated in this study

		Before		After	
Sex [*n*(%)]	Female	24 (64.9)		24 (64.9)	
	Male	13 (35.1)		13 (35.1)	
Operation type [*n*(%)]	1st	17 (45.9)		17 (45.9)	
	2nd	20 (54.1)		20 (54.1)	
Blood pressure [*n*(%)]	Normal	4 (10.8)		9(24.3)	
	Elevated	6 (16.2)	High 33(89.2)	6 (16.2)	High 28 (78.7)
	High (stage 1)	7 (18.9)		1 (2.7)	
	High (stage 2)	17 (45.9)		21 (56.8)	
	Hypertensive Crisis	3 (8.1)		0 (0)	
Salivary alpha-amylase score [*n*(%)]	Score 1	34 (91.9)		29 (78.4)	
	Score 2	2 (5.4)	High 3 (8.1)	1 (2.7)	High 8 (21.6)
	Score 3	0 (0)		6 (16.2)	
	Score 4	1 (2.7)		1 (2.7)	
Heart rate [mean ± SD]		79.2 ± 11.9		75.2 ± 11.9	
Oxygen level [mean ± SD]		96.9 ± 1.6		97.4 ± 1.3	

**Fig. 1:**
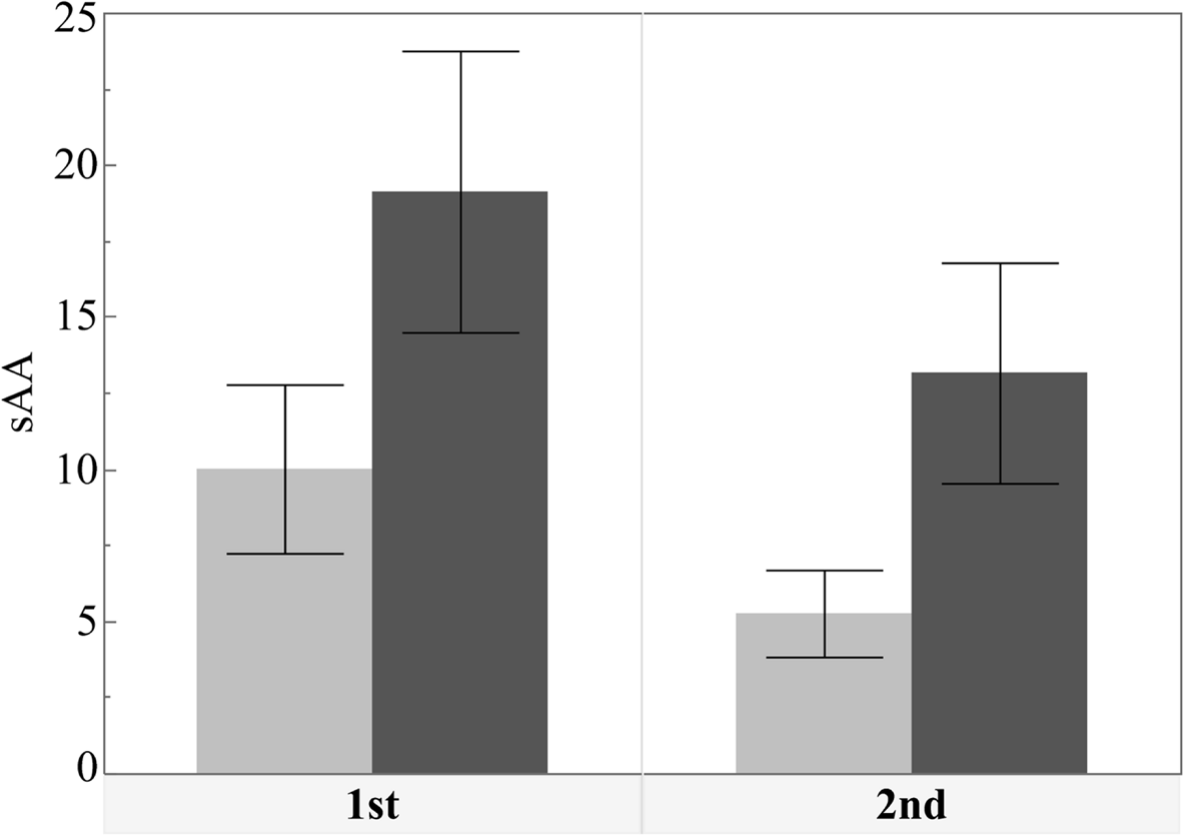
Bar chart showing the mean sAA activity level before and after operation. A significant increase was seen in sAA after the operation (*p*<0.001)

**Fig. 2:**
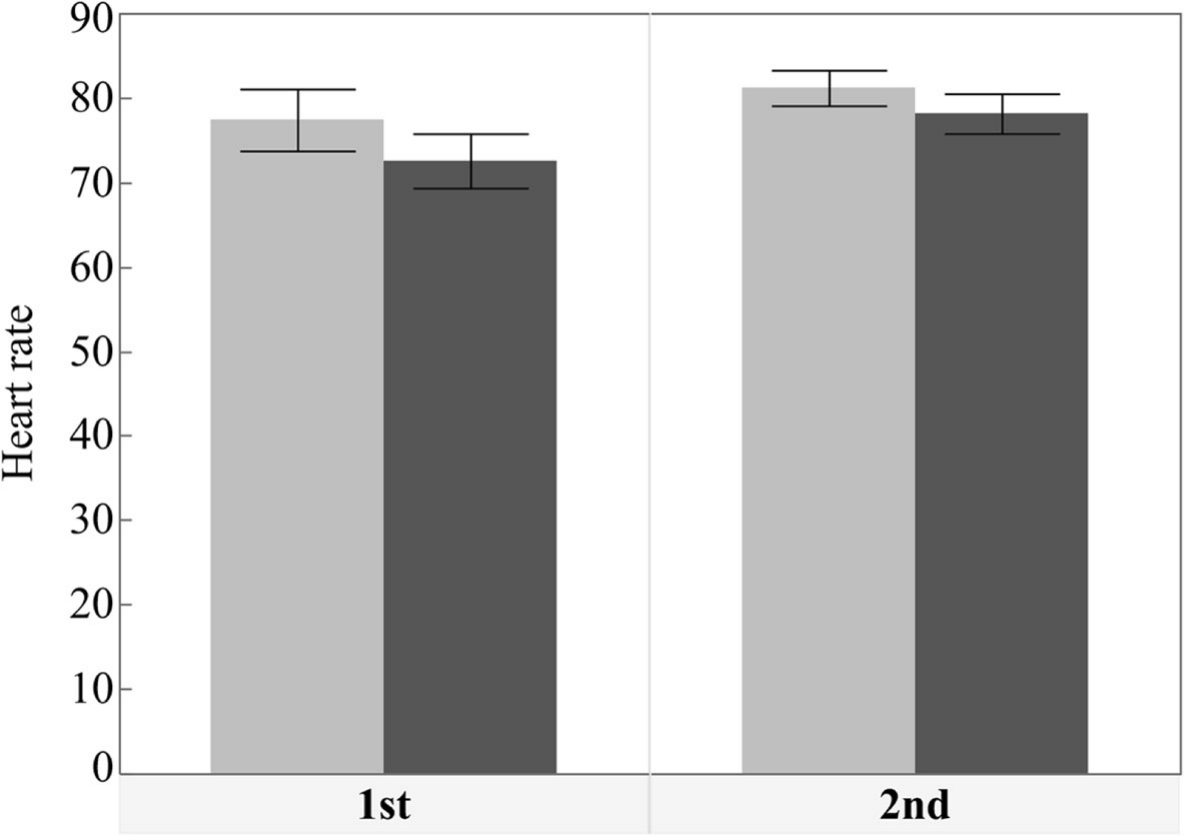
Bar chart showing the mean HR scores before and after operation

**Fig. 3:**
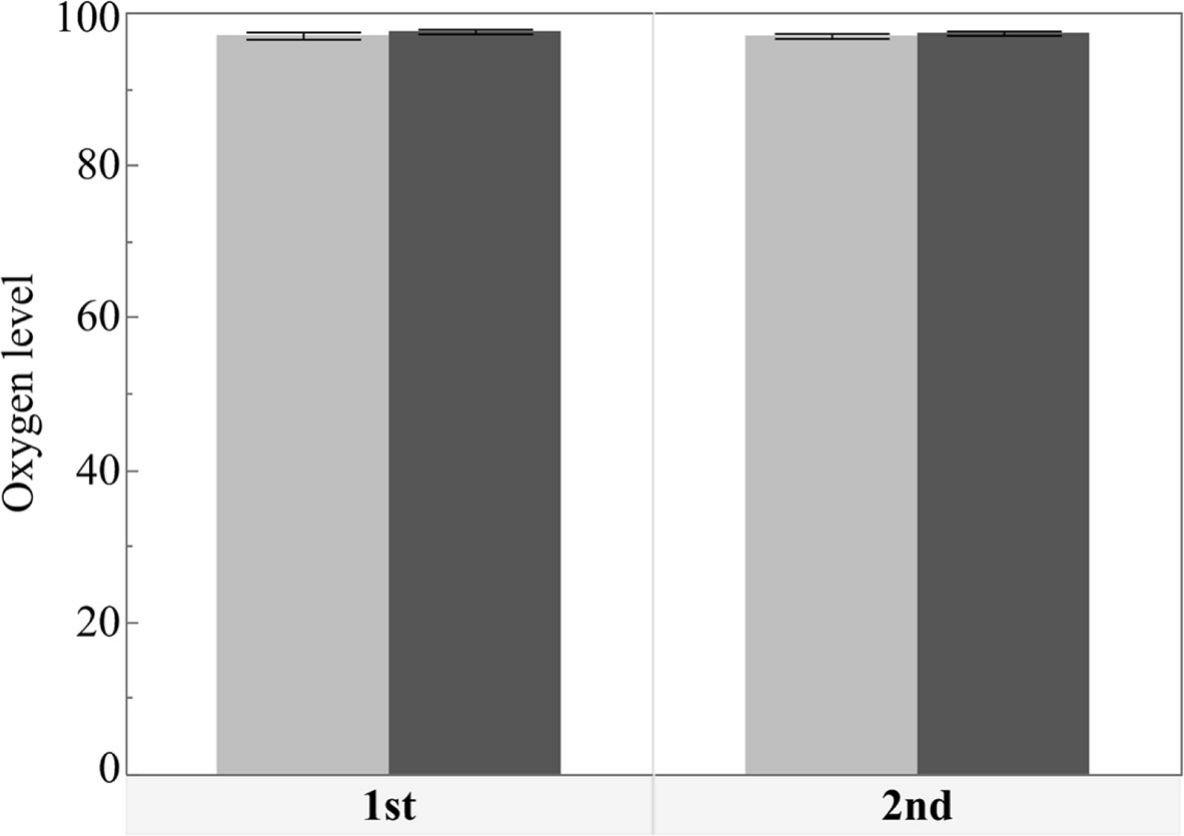
Bar chart showing the mean SpO_2_ scores before and after operation

A significant increase was observed in sAA after implant surgery (*p*<0.001). On the other hand, the changes in the postoperative HR and SpO_2_ values were not significant (*p*=0.073 and 0.102, respectively). Only the systolic BP decreased significantly after surgery (*p*=0.001) (Figs. 1, 2 and 3, Table 3).

**Table 3:** Paired comparison between the different parameters before and after surgery

	Before	After	Difference	*p* value
sAA	10.8 ± 22.5	18.5 ± 23	−7.7 ± 33.5	<0.001*
HR	79.2 ± 11.9	75.2 ± 11.9	5.4 ± 17.9	0.073
SpO_2_	96.9 ± 1.6	97.4 ± 1.3	4.7 ± 12.3	0.102
BP (systolic)	141.5 ± 19.6	136.1 ± 18.3	4 ± 6.6	0.001*
BP (diastolic)	82.1 ± 13.5	77.4 ± 11.8	−0.4 ± 1.5	0.084

*Significant

BP: blood pressure, sAA: salivary alpha-amylase, HR: heart rate, SpO2: peripheral blood oxygen saturation

**Fig. 4:**
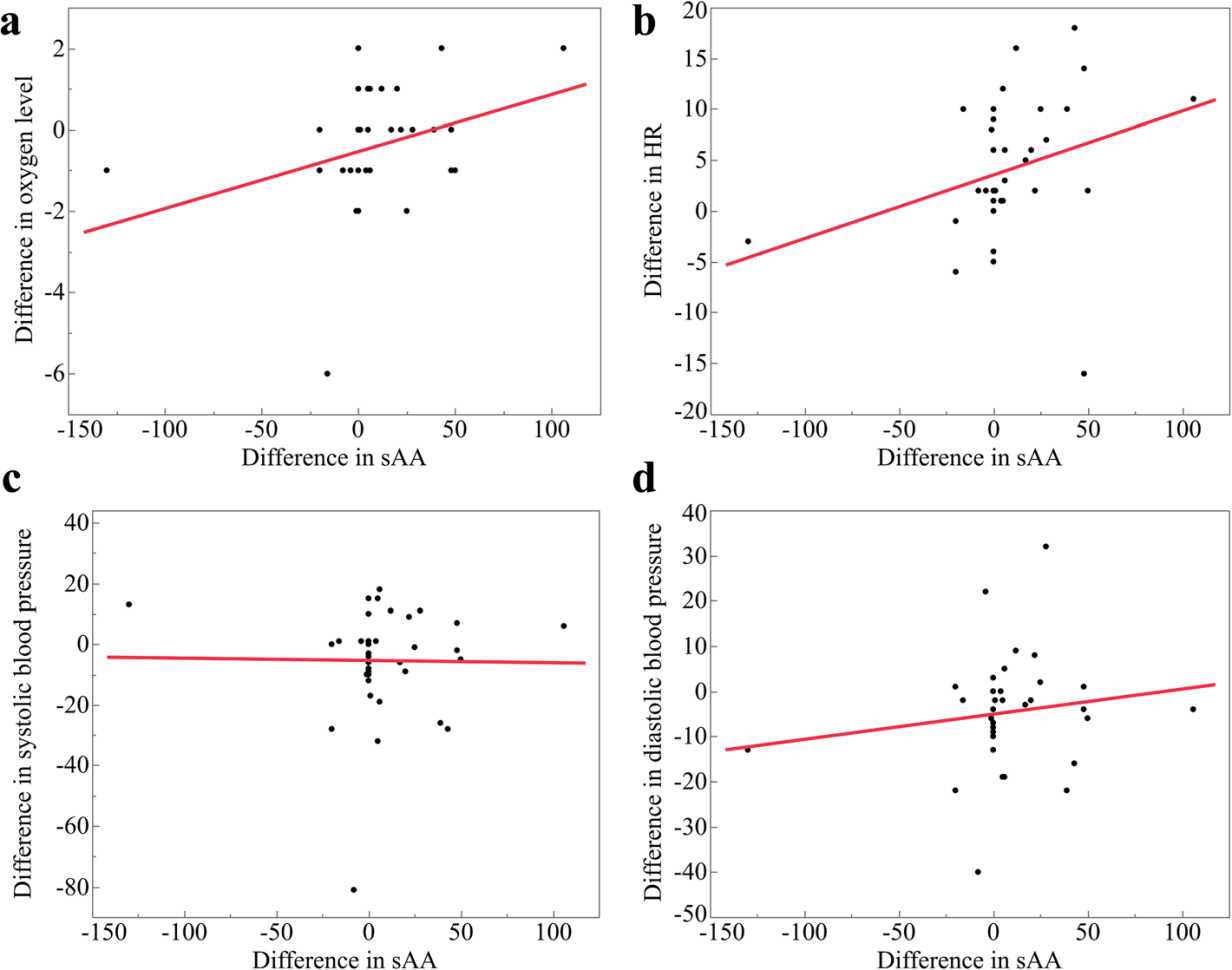
Scatter plot showing the correlation between the difference of sAA and **a** oxygen level, **b** heart rate, **c** systolic blood pressure, and **d** diastolic blood pressure. A positive correlation presented between difference in sAA and **a** oxygen level and **b** heart rate (*p*<0.05)

A significant positive correlation was observed between sAA and HR (*r*_s_=0.434, *p*=0.007) (Fig. 4b). A significant positive correlation was also observed between sAA and SpO_2_ (*r*_*s*_=0.392, *p*=0.016) (Fig. 4a). Conversely, the correlation between sAA and BP was insignificant (systolic; *r*_*s*_=0.078, *p*= 0.646, diastolic; *r*_*s*_=0.195, *p*=0.247) (Fig. 4c, d, Table 4).

**Table 4:** Correlation between the differences in the observed parameters after surgery

		HR	SpO_2_	BP (systolic)	BP (diastolic)
sAA	Correlation coefficient	0.434	0.392	0.078	0.195
	*p* value	0.007*	0.016*	0.646	0.247

^*^Significant

HR: hear rate, sAA: salivary alpha-amylase, BP: blood pressure, SpO2: peripheral blood oxygen saturation

Eight patients in this study exhibited high sAA activity on the follow-up measurement (i.e., after surgery), compared to only three patients who exhibited high sAA activity before surgery. This may be indicative of the increase in the patients’ stress levels during surgery, which would be related to the surge in sAA activity.

## Discussion

The activity of sAA is affected by several factors, which mainly include physical and psychological stressors. A study has reported that sAA activity increases in response to the acute stress induced by venepuncture during a periodic medical examination and remains elevated 15 min after the procedure [21]. Another study reported significant differences in sAA, salivary cortisol, plasma catecholamines, and cardiovascular parameters between the stress and resting conditions [17]. Ali et al. reported that sAA activity was elevated during and even 15 min after venepuncture [21], even though venepuncture during periodic occupational health examinations is less invasive than the procedures used in dental implant-related surgeries. Similarly, our study found that sAA activity was elevated after surgery, even though the other vital signs returned to normal immediately. Thus, the results suggest that sAA activity is elevated because of the fear of pain or stress, and the elevation can last long enough to enable subsequent monitoring. This implies that sAA activity can be used as an additional method to monitor the patient’s history of mental and physical stress or the accumulation of such stress perioperatively.

Invasive major surgeries are usually performed under general anesthesia, which means that the patient is unconscious and does not experience fear of pain or mental stress during the procedure, irrespective of the operative time, whereas the patient must keep his/her mouth open during dental surgery, and the sound and sensation of drilling in the jawbone may cause a certain level of stress or fear since the patient is completely or partially conscious. Additionally, the patients cannot see their oral cavity during these procedures, which may induce severe anxiety in some individuals. We opine that evaluating the patient’s anxiety fosters improvement in the preoperative informed consent taking activity and the surgical environment.

In this study, sAA activity was elevated predominantly in the postoperative measurement, which may be attributed to several factors. The duration of surgery may be one such factor, which may affect the stress level of the patient. Another factor may be related to patient exhaustion: if the surgery is long, the patient may get tired, causing the stress level to rise [22]. Furthermore, the proportion of patients with high sAA activity (>31: somewhat stressed level in accordance with Table 5) was greater after surgery than that before surgery. This may be representative of the surge in the patient’s stress level during surgery, which would be related to an increase in sAA activity. We also observed a significant correlation between sAA activity and HR, indicating that it may be a valuable biomarker for evaluating stress and anxiety in dental patients [23].

**Table 5:** Reference values for salivary alpha-amylase

Results (kIU/L)	Stress level
0–30	None
31–45	Somewhat stressed
46–60	Stressed
>61	Very stressed

One of the patients developed a hypertensive crisis, which led to cancellation of surgery and exclusion of data from the final analysis. We measured his sAA activity, which was 69 kIU/L (very stressed, based on Table 1). We assumed that this patient was very stressed, and his BP and sAA activity may have been correlated and increased due to anxiety and stress [5]. However, our study did not find any statistically significant correlation between sAA and BP.

We could not determine whether intravenous or local anesthesia had any effect on sAA, owing to the limited sample size of our study. However, most patients who underwent intravenous anesthesia exhibited an increase in the sAA activity during the postoperative measurement. Furthermore, our results showed an increase in the overall postoperative measurement of SAA activity. No difference was detected with respect to the type and duration of surgery.

In our study, the operative time was higher for the first surgery (median=56.5 and range=35 min) compared to the second surgery (median=27 and range=15.8 min). The operative time was excluded from comparison, as was the comparison between the first and second surgeries. Our principal aim was to monitor sAA activity and its correlation with other measurable variables as a general effect, irrespective of other variables.

Further studies are required to evaluate the difference in sAA activity caused by the operative time, surgical procedure, sex-based differences, and types of anesthesia.

## Conclusion

This study found a significant positive correlation between sAA activity and HR; furthermore, SpO_2_ and sAA activity also showed a significant positive correlation.

Our results found no correlation between BP and sAA. Finally, sAA activity may provide additional information to monitor the history of the patient’s mental and physical stress or the accumulation of such stress during dental implant surgery.

## Data Availability

The datasets used and/or analyzed during the current study are available from the corresponding author on reasonable request.

## Abbreviations

ANS: Autonomic nervous system
LA: Local anesthesia
IV: Intravenous anesthesia
PSNANS: Parasympathetic nervous system
sAA: Salivary alpha-amylase
SNS: Sympathetic nervous system
SpO_2_: Peripheral blood oxygen saturation
